# Dissolution of simulated nuclear waste glass at high surface area to solution volume, high pH and 70 °C: comparison of international simple glass and SON68 glass

**DOI:** 10.1039/d4ra04936e

**Published:** 2024-11-04

**Authors:** Felix Brandt, Sébastien Caes, Martina Klinkenberg, Juri Barthel, Sanheng Liu, Karel Lemmens, Dirk Bosbach, Karine Ferrand

**Affiliations:** a Institute of Fusion Energy and Nuclear Waste Management (IFN-2), Forschungszentrum Jülich GmbH 52425 Jülich Germany f.brandt@fz-juelich.de; b Institute of Sustainable Waste & Decommissioning, SCK CEN B-2400 Mol Belgium; c Ernst Ruska-Centre (ER-C 2), Forschungszentrum Jülich GmbH 52425 Jülich Germany

## Abstract

Long-term static dissolution experiments, lasting up to ∼1500 days, were conducted on International Simple Glass (ISG) and SON68 glass under hyperalkaline pH, at 70 °C, and at a very high glass surface area to solution volume ratio. The study compared (1) glass dissolution kinetics, (2) secondary phase formation, and (3) the microstructure of the altered glass and secondary phase interface. Boron release indicated rapid initial dissolution followed by a slowdown mainly due to a significant pH drop. ISG reached a residual rate regime, while SON68 approached this regime near the experiment's end, with both glasses having similar final dissolution rates. Electron microscopy (SEM, TEM, EDS) of the reacted glass surfaces and the alteration products revealed nontronite formation on SON68, while C(A)SH phases and later rhodesite appeared on ISG, in addition to phillipsite-type zeolite formation observed in both experimental series. TEM observations revealed a porous, foam-like surface altered layer (SAL) near the pristine glass. SON68's SAL nanostructure, more complex than ISG's, had two porous zones, hindering water transfer and glass constituent release, in addition to a pH drop reducing silica network hydrolysis. TEM-EDS showed cation exchange and iron depletion in SON68's SAL, leading to nontronite formation. Secondary phases at the SAL-solution interface did not destabilize the SAL, and no alteration resumption was observed due to the pH drop below the threshold necessary for an alteration resumption due to zeolite formation. In conclusion, the combination of alkaline conditions and very high reaction progress does not lead to the dissolution of the glass by a dissolution-reprecipitation mechanism, as typically observed at much lower SA/V ratios. At the relatively mildly alkaline pH reached within the first year of the experiments, the diffusion of cations through the SAL becomes rate-controlling.

## Abstract 2

Long-term dissolution experiments on two nuclear waste glasses indicate a diffusion-controlled dissolution mechanism including the formation of a porous surface altered layer and secondary phases without a resumption to higher rates.

## Graphical abstract

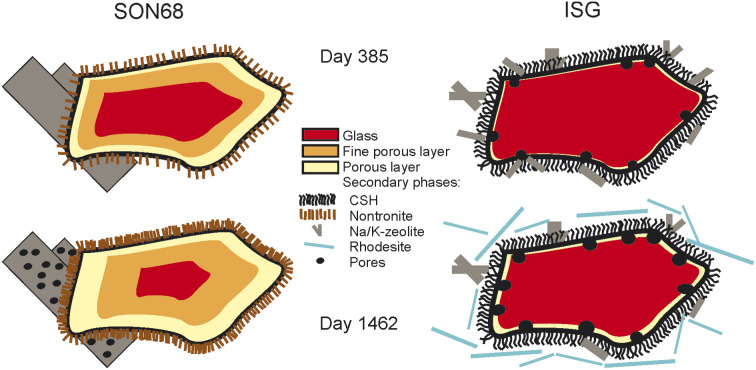

## 1 Introduction

Reprocessing of spent nuclear fuel and vitrification of the resulting high- and intermediate-level effluents are common steps that are included in the closed nuclear fuel cycle.^1–4^ The storage of the vitrified waste packages, consisting of glass and stainless-steel containers, in deep geological repositories (DGRs) is currently considered as the preferred disposal option.^5–7^ Most nuclear waste glasses are borosilicate glasses, due to the high chemical durability and possible waste loadings in the range between 15 and 30 wt% oxide.^1,8–10^

After disposal of the nuclear waste glass, the alteration of the borosilicate glass by aqueous solution leads to the release of radionuclides. The alteration involves several serial and parallel processes,^11–13^ which include two main mechanisms of chemical attack: (1) the exchange between the mobile ions of the glass network and the protons or hydronium ions and (2) the hydrolysis of the silicate network, which leads to the formation of silanol groups and the release of orthosilicic acid.^10,11,14,15^ Typically, three kinetic regimes of glass alteration are distinguished: (1) the ‘initial rate’ regime where glass dissolves at the highest rate (Stage I), (2) the ‘residual rate’ regime with a rate typically orders of magnitude lower than in the ‘initial rate’ regime (Stage II), (3) the ‘alteration resumption’ regime (Stage III).^16–18^

On a microscopic scale, the ‘residual rate’ regime is often linked to the formation of a passivating porous Si-rich surface alteration layer (SAL) by silica precipitation on the glass surface and condensation of the silanol groups with other elements coming from the glass dissolution or supplied by the leaching solution.^19^ The drop from initial to residual rate is attributed to the combination of a thermodynamic and a transport limiting effect.^20–22^ Microscopically, the SAL often appears to consist of aggregated Si colloids which sometimes form layers.^23–25^ According to Gin *et al.*,^26^ at neutral pH, this description by dissolution-reprecipitation may be less applicable, and the SAL may be formed more by local rearrangement. The transport of the aqueous species by diffusion through the SAL is slowed down, thus decreasing the glass dissolution rate.

The resumption of glass alteration usually coincides with the precipitation of secondary phases at the SAL-solution interface, which can destabilize the SAL by extraction of a fraction of the network-forming elements, *i.e.*, mostly Si and Al.^18,21,27^ At conditions typical for the common standardized glass dissolution tests (*e.g.* MCC-1 static leach tests [ASTM C1220]^28^ or Product Consistency Tests [ASTM C1285]^29^), *i.e.* near neutral starting pH and 90 °C, the ‘residual rate’ regime appears to be a stable state, whereas resumption of glass dissolution typically occurs at high pH and/or temperature.^17,30^

To improve the mechanistic understanding of its formation and to investigate its structure, the SAL has been probed with high resolution methods, *e.g.* transmission electron microscopy (TEM), secondary ion mass spectrometry (nano-SIMS), or atom probe tomography (APT),^31–33^ typically on samples obtained from experiments at near neutral starting pH and low glass surface area to solution volume ratio (SA/V). Essentially three models have been proposed to explain the mechanisms of the SAL formation, which are highly dependent on the experimental conditions, (1) the recondensation model, (2) the dissolution-reprecipitation model, and (3) a combination of the first two models to an intermediate model.

(1) The recondensation model suggests a preferential leaching of the weakly bonded elements. In this model, the in-diffusion of water species (hydration), ion-exchange reactions between protons and network modifying or charge compensating glass species such as the alkali metals and their out-diffusion through the SAL are supposed to control the glass dissolution kinetics.^15^ As the rate of interdiffusion initially exceeds the rate of silicate network hydrolysis, a hydrated glass layer without the mobile glass species is formed. The concentration of network formers in solution increases, eventually leading to a steady state. This may be seen as a saturation state of the aqueous solution with respect to the SAL in a first approximation.^11,18,34^ Hydrolysis of the glass network decreases with time due to the development of a transport limiting gel-layer or SAL, which is assumed to form *via* condensation reactions of the hydrated relict glass structure. Saturation (affinity) effects and transport factors are then seen responsible for the drop in the dissolution rate towards the residual rate.^35^

(2) In the dissolution-reprecipitation model, the SAL is supposed to be formed after congruent glass dissolution and precipitation of the less soluble elements.^32^

(3) An intermediate model in which partial hydrolysis of Si species occurs, followed by *in situ* condensation, has also recently been described. This new concept can be seen as the more general glass dissolution model including the ideas of the first two models as extreme cases. All intermediate mechanisms may be possible as well, even within one dissolution test.^18^

In the absence of external pH buffers, boron and sodium oxides will largely determine the pH of the leaching solution in contact with the nuclear glass within the pH region between 9 and 10.^36,37^ However, when cementitious materials are used as backfill, linings or plugs in a DGR, much higher pH values of the (leaching) solution could prevail upon first contact with the glass matrix.^38–40^ Müller *et al.*^41^ observed zeolites and clay minerals as secondary phases and a thick alteration layer after leaching soda-lime boroaluminosilicate glass at low SA/V and high pH. However, only a few studies have addressed the specific case of cementitious materials dominating the solution chemistry in combination with a very high SA/V ratio, which allows investigating the advanced reaction progress stage. Recently, the alteration of the International Simple Glass (ISG) in a synthetic cementitious water (young cement water + Ca, YCWCa) at 70 °C and two different SA/V ratios (*i.e.* 8240 m^−1^ and 264 000 m^−1^) has shown the formation of a gel-like SAL, Calcium Silicate Hydrate phases (CSH) and zeolites, but no glass alteration resumption.^25,42^ Although the ISG dissolution rates determined from the boron normalized mass loss were quite similar, the microstructure of the SALs, as observed in these experiments, varied significantly. At SA/V of 8280 m^−1^, a typical layered structure of a colloidal SAL up to several micrometers thick was visible, whereas at SA/V of 264 000 m^−1^ only a very thin SAL of 80–250 nm thick was observed, covered by CSH phases. Within the time scale of the experiments, a resumption of glass alteration was not observed in any of the experiments.

Very thin SALs were also reported in earlier publications.^43^ So far, they have not been systematically studied on nuclear waste glasses, although the conditions at which they have occurred are relevant for DGR assessment. In this paper, the long-term alteration of the complex SON68 glass in YCWCa at a very high SA/V, high pH and 70 °C is compared with that of ISG to assess the effect of glass composition upon dissolution. In addition to the determination of the dissolution rates, a detailed characterization of the SAL on the nanoscale was carried out in combination with an identification of the secondary phases. A focus was set on the later stage of glass dissolution up to ∼1500 days.

## 2 Materials and methods

### 2.1 Glass samples

Samples of two model systems for nuclear waste glass were used in the experiments: the inactive French nuclear glass SON68 produced by CEA (R7T7 – Lot number: 1865) and the International Simple Glass (ISG) produced by MoSci Corporation (Rolla, MO, USA – Lot number: L12012601-M12042403). The nominal composition of both glasses as provided by the suppliers is given in Table 1. Glass powders were prepared as described in Ferrand *et al.*^42^ by milling and sieving to the grain size fraction between 20 and 25 μm and removing ultrafine particles by ultrasonic cleaning. The BET (Kr) specific surface area was determined to be 0.504 ± 0.002 m^2^ g^−1^ for SON68 glass and 0.440 ± 0.002 m^2^ g^−1^ for ISG.

**Table 1:** Nominal composition of SON68 and ISG glasses in wt%

Oxide	SON68	ISG
SiO_2_	45.50	56.20
B_2_O_3_	14.00	17.30
Na_2_O	9.86	12.20
Al_2_O_3_	4.91	6.06
CaO	4.04	4.98
Fe_2_O_3_	2.91	—
ZrO_2_	2.65	3.28
ZnO	2.50	—
Li_2_O	1.98	—
MoO_3_	1.70	—
Nd_2_O_3_	1.59	—
Cs_2_O	1.42	—
Ce_2_O_3_	0.93	—
La_2_O_3_	0.90	—
NiO	0.74	—
MnO_2_	0.72	—
Traces <0.7 wt% of: Ba, U, Cr, Pr, Sr, Th, P, Te, Y, Co, Ag, Cd, Sn, Sb		—

### 2.2 Dissolution experiments

Batch dissolution experiments were carried out in the same setup as described in Ferrand *et al.*^42^ The experimental conditions are summarized in Table 2. The experiments consisted of Teflon® containers with a volume of 20 mL, into which 3 g of glass powder and 5 g of the same batch of YCWCa solution, prepared as described in the data publication,^44^ were added.

**Table 2:** Summary of the experimental conditions for the batch dissolution experiments

Parameter	Setting
Temperature (°C)	70 ± 1
Particle fraction (μm)	20–25
Mass of glass powder (g)	3.000 ± 0.005
Specific surface area of glass powder (m^2^ g^−1^)	
ISG	0.440 ± 0.002
SON68 glass	0.504 ± 0.002
pH of YCWCa solution	pH (70 °C) = 12.5 ± 0.2
Weight of YCWCa solution (g)	5.000 ± 0.005
SA/V (m^−1^)	
ISG	264 000 ± 2600
SON68	302 400 ± 3000

Each data point/sample date discussed later represents a separate experiment, which was started at the same time as the other experiments, using the same batch of glass powder of SON68 glass or ISG and the same YCWCa solution. The data were corrected for possible evaporation by checking the weight of the Teflon® containers frequently.

The containers were kept under argon at 70 ± 1 °C in an oven placed in a glove box. The suspension was homogenized manually once a week with a Teflon® coated stirring bar, which was placed inside the container, without opening the container.

The chemical conditions of the experiments were determined by the YCWCa solution, which is characterized by a pH (70 °C) of 12.5 and the chemical composition as summarized in Table 3. Individual batch experiments were ended after 59, 288, 385, 632, 952 and 1462 days. The experimental data of ISG of days 59, 288, 385, 632, and 952 were already reported in Ferrand *et al.*^42^ and are used here for comparison with new corresponding data for SON68. In addition, to enable a comparison with the SON68 experiments, a parallel ISG experiment was performed and sampled after 1462 days. At the end of each experiment, the container was removed from the oven, shaken for homogenization of the suspension, and allowed to cool down to room temperature for two hours for sampling. After weighing of the container and settling of the glass particles, the container was opened under argon and a needle attached to a syringe was used to collect the solution. The pH was measured at room temperature.

**Table 3:** Chemical composition of young cement water containing Ca

Element	Concentration (mg L^−1^)
Al	0.06 ± 0.04
B	<1
Ca	17.8 ± 1.8
Na	3120 ± 310
K	12 400 ± 1200
Si	0.48 ± 0.21

Then, the solution was filtered through a 0.45 μm membrane filter (GHP acrodisc from PALL). From this sample, an aliquot of 1 mL of solution was diluted with 2 mL of Milli-Q^®^ water (18.2 MΩ cm at 25 °C) and ultrafiltered (10 kD, Microsept TM, advance from Cytiva) at 5000 rotations per minute (RPM) for 20 minutes and analyzed by ICP-OES (IRIS Intrepid II dualview, Thermo Scientific, USA) and ICP-MS (XSERIES2, Thermo Scientific, USA). For the measurements, the sample was diluted and acidified with either 5% HCl and 1% HNO_3_ (ICP-OES) or 2% HNO_3_ (ICP-MS), respectively.

The altered glass powder was collected in a polypropylene container at the end of each experiment and rinsed with Milli-Q^®^ water. The excess water was removed using a syringe with a needle and the container was placed in an oven at 30 °C until a constant weight was reached. A minimum of five measurements was carried out.

The dried altered glass powder was kept in a desiccator. For the investigation of the solids and focused ion beam (FIB) preparation, a subsample of each powder was embedded in an epoxy resin (Struers, Germany) and polished with diamond suspensions (9 μm, 3 μm and 1 μm, respectively).

### 2.3 Calculation of normalized mass loss and dissolution rate

The release of boron is regarded as an indicator for the mass loss of the glass because boron is not supposed to be retained in the secondary phases or in the SAL.

The normalized mass loss of the glass at a given time, NL(B) (g m^−2^), is calculated based on the boron release according to eqn (1):1
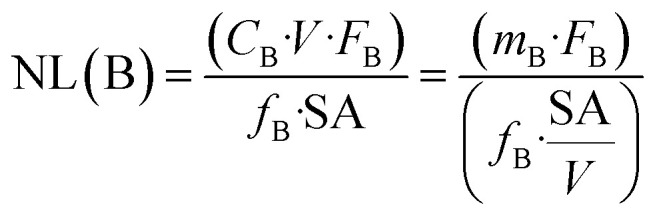
where *C*_B_ is the boron concentration in the aliquot of solution (g m^−3^), *V* is the total volume of solution (m^3^), *F*_B_ is the factor to convert the atomic weight of the element B to the molecular weight of B_2_O_3_, *f*_B_ is the weight percentage of B_2_O_3_ in the pristine glass, SA is the total surface area of the exposed glass (m^2^) and *m*_B_ is the mass of B (g).

The glass dissolution rate (*r*) is derived from the normalized mass loss of boron, NL(B) according to eqn (2):

2 *r* = d(NL(B))/d*t*

### 2.4 X-ray diffraction (XRD)

The grain size fraction <5 μm was separated *via* sedimentation according to the procedure of Ferrand *et al.*,^42^ which leads to an enrichment of the crystalline secondary phases. For the XRD measurements, a small amount of powder of the separated grain size fractions was dispersed in 1 mL Milli-Q^®^ water and deposited on a zero-background silicon single crystal wafer. Then, the samples were dried at 40 °C on a heating plate. The XRD measurements were carried out with a D4 Endeavor diffractometer (Bruker AXS GmbH, Karlsruhe, Germany) in Bragg–Brentano geometry using Cu radiation (CuK_α1_ = 1.5405 Å) at 40 mA and 40 kV. The instrument is equipped with a linear silicon strip LynxEye detector (Bruker-AXS, Karlsruhe, Germany). XRD patterns were collected in the range from 5° to 100° 2*Θ* using a step size of 0.01°/2*Θ* and a counting time of 10 s/step at ambient conditions.

### 2.5 Scanning (SEM) and transmission electron microscopy (TEM)

The evolution of the glass alteration and formation of secondary phases were studied using the environmental scanning electron microscope Quanta 200 F (FEI, Eindhoven, The Netherlands) in low vacuum mode at 60 Pa. Additionally, a Phenom scanning electron microscope (Phenom PRO X, Thermo Fisher Scientific, The Netherlands) was used at an accelerating voltage of either 5 kV or 15 kV.

The NVision 40 cross beam station (Carl Zeiss AG, Germany) was used for the preparation of thin cross-section lamellae by focused ion beam milling (FIB). The procedure is described in detail in Lenting *et al.*^33^ and Ferrand *et al.*^42^

Scanning Transmission Electron Microscopy (STEM) and energy dispersive X-ray spectroscopic (EDS) elemental mapping were carried out with a TFS Spectra 300 (Thermo Fischer Scientific, The Netherlands) operated at 200 kV accelerating voltage and with a 230 pA beam current. The focused STEM probe was formed with a convergence semi-angle of 27.5 mrad and corrected for spherical aberration. High-angle annular dark-field (HAADF) images were recorded with a detector covering scattering angles between 62–200 mrad, realizing Z-contrast imaging of the thin cross section, where higher intensity indicates a composition with higher atomic number, larger sample thickness, or higher material density.^45^ Energy dispersive X-ray mappings were acquired while scanning using a Super X EDS detector (Thermo Fischer Scientific, The Netherlands)^46^ with 0.7 sr maximum collection angle to determine qualitatively the elemental distribution in the SAL and the surrounding material. The spectrometer was operated with a dispersion of 5 eV per channel covering X-ray energies up to 20 keV and the readout was synchronized with the scan using a dwell time of 10 μs. Total acquisition times for the mappings were between 20 and 30 minutes and empirical background correction was applied. About one quarter of the detector area was shadowed by the sample holder, so the effective solid angle for EDS detection was approximately 0.5 sr.

## 3 Results and discussion

### 3.1 Solution analyses

#### 3.1.1 Evolution of pH and general dissolution

**Fig. 1:**
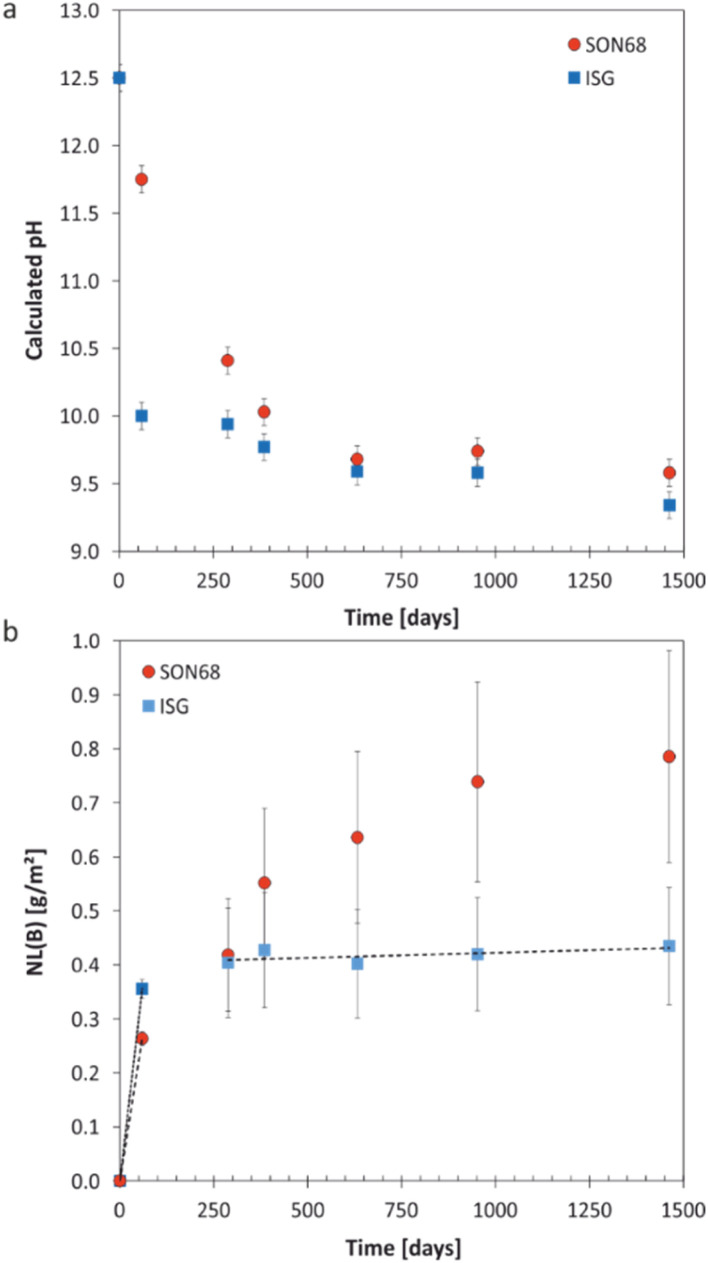
(a) Evolution of calculated pH at 70 °C with time for SON68 glass and ISG in YCWCa at 70 °C; (b) normalized mass loss of boron NL(B) of SON68 and ISG up to 1462 days including the linear regressions from which the dissolution rates were derived (dotted line: initial dissolution rate of SON68 glass: 4.4 × 10^−3^ g per m^2^ per day, dashed lines: initial and residual dissolution rate of ISG: 6 × 10^−3^ g per m^2^ per day and 2 × 10^−5^ g per m^2^ per day, respectively). Data for ISG plotted for comparison are taken from Ferrand *et al.*,^42^ except for day 1462.

**Fig. 2:**
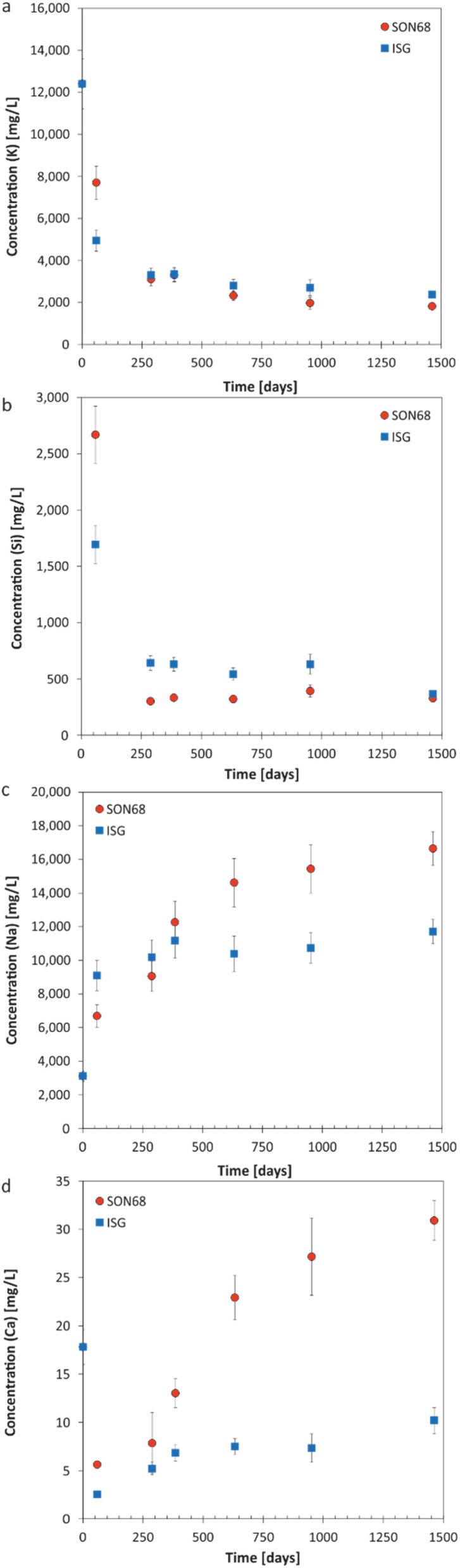
Evolution of element concentration with time in solution for SON68 and ISG in YCWCa at 70 °C (a) K; (b) Si; (c) Na; (d) Ca. Data for ISG are taken from Ferrand *et al.*^42^ except for day 1462.

The pH measured at room temperature was calculated to the experimental temperature of 70 °C by considering the increase of the water dissociation constant (*K*_w_) with increasing temperature, *i.e. K*_w_ = 1.58 × 10^−13^ at 70 °C (Fig. 1a). All dissolution experiments were started at pH = 12.5 ± 0.2 (corresponding to a pH 13.7 measured at 25 °C). The initial fast drop of pH was mainly due to the dissolution of the glass network formers such as SiO_2_ and B_2_O_3_, as evidenced by the initial fast increase of B and Si in the solution (Fig. 1b and 2b). The pH of the SON68 experimental series slowly approached a pH of 9.6 between day 385 and day 632, whereas in the parallel ISG dissolution experiments a pH drop to a value of about 10 was already reached within the first 59 days. The pH of both experimental series at the sampling points after day 385 was similar, with a slightly decreasing pH towards the end of the experiments, remaining within a similar range of pH = 9.3–9.6.

The evolution of the normalized mass loss of boron NL(B), *i.e.*, of the total amount of glass dissolved at the time of each sampling point based on the boron release in solution, is shown in Fig. 1b. From the NL(B), initial dissolution rates of 6 × 10^−3^ g per m^2^ per day and 4.4 × 10^−3^ g per m^2^ per day were calculated for ISG and SON68 glass for the first 59 days, respectively. The boron normalized mass loss and pH are linked at the early stages of the experiments *i.e.*, the earlier pH drop observed for ISG corresponds with an earlier approach to a plateau of NL(B). For ISG, this plateau can be described by a linear fit (indicated by a dashed line in Fig. 1b), resulting in a residual rate of 2 × 10^−5^ g per m^2^ per day. At the very end of the experiments, the SON68 glass dissolution rate also seems to approach a plateau, and a ‘residual’ rate of 9 × 10^−5^ g per m^2^ per day was calculated from the last two data points. At all times after day 288, the SON68 glass appears to dissolve at a significantly higher rate than ISG. At day 1462, the cumulative amount of dissolved glass based on NL(B) was about 0.78 g m^−2^ for SON68 *versus* 0.44 g m^−2^ for ISG.

#### 3.1.2 Element release during glass alteration

The aqueous element concentrations of K, Si, Na, and Ca measured for ISG and SON68 are shown in Fig. 2 and summarized in the data publication^44^ related to this article. The initial concentration of K of 12 400 mg L^−1^ is set by the composition of the YCWCa solution as K is not present in ISG or SON68. The decreasing concentration of K (Fig. 2a) with time is related to cation exchange and secondary phase formation, as discussed later in Section 3.2. At the early stage of the experiments, a higher concentration of K in solution was observed for SON68 than for ISG, but in the samples of ≥288 days the K concentrations in solution were similar for both glasses at about 2000 mg L^−1^.

The evolution of the Si concentration in solution (Fig. 2b) follows a similar trend to the K concentration (Fig. 2a), suggesting the Si retention in a solid phase. Most significant is the difference between ISG and SON68 series during the initial regime of the experiments. On day 59, a Si concentration of about 2700 mg L^−1^ was measured in the SON68 experiment, while a Si concentration of only 1700 mg L^−1^ was reached in the ISG experiment. After day 288, the Si concentration in the SON68 experiment decreased down to about 370 mg L^−1^ and stayed constant, within the experimental uncertainty, until the end of the experiments. For ISG, a Si steady state concentration at about 650 mg L^−1^ was reached after day 288 but was not maintained until the end of the experiment, as at the last data point it decreased to a concentration like the one measured for SON68.

Na is present in the YCWCa solution as well as in the glass samples. The Na concentration increased with time in both experiments, following a trend similar to NL(B) (Fig. 1b and 2c). Although ISG contains more Na than SON68 (Table 1), less Na was measured in the ISG experiment after 1462 days, revealing less alteration of ISG than SON68 and/or more Na retention in the SAL and/or secondary phases in the ISG experiment. After day 288, a concentration plateau for Na appeared for ISG at about 10 000–12 000 mg L^−1^. For SON68, after day 288 the Na concentration in solution continuously increased up to about 16 500 mg L^−1^ at the end of the experiment. Significantly more Na was released into solution than K taken up in the SAL and/or secondary phases in both experimental series. This indicates an exchange of Na for K, as later discussed in detail in Section 3.2, in addition to Na release by congruent dissolution and exchange with H_3_O^+^.

Ca was present in both glass types as well as in the YCWCa solution. Starting from 18 mg L^−1^, the concentration of Ca dropped in both experimental series to much lower values, which indicates Ca retention in the SAL and/or secondary phases (Fig. 2d). For ISG, the Ca concentration remained stable between 385 and 952 days at about 7 mg L^−1^, and then increased up to about 10 mg L^−1^ at day 1462, suggesting the destabilization of the glass alteration layer. Ca concentrations in the SON68 series were generally higher than in the ISG series and continuously increased up to the final sampling to values of more than 30 mg L^−1^ – higher than present in the initial YCWCa solution.

### 3.2 Characterization of the solids

Glass samples taken after 385 and 1462 days were analyzed to investigate whether and how SON68 and ISG glass surface and internal structure, as well as secondary phases changed during the residual rate regime.

#### 3.2.1 Identification of secondary phases by XRD

**Fig. 3:**
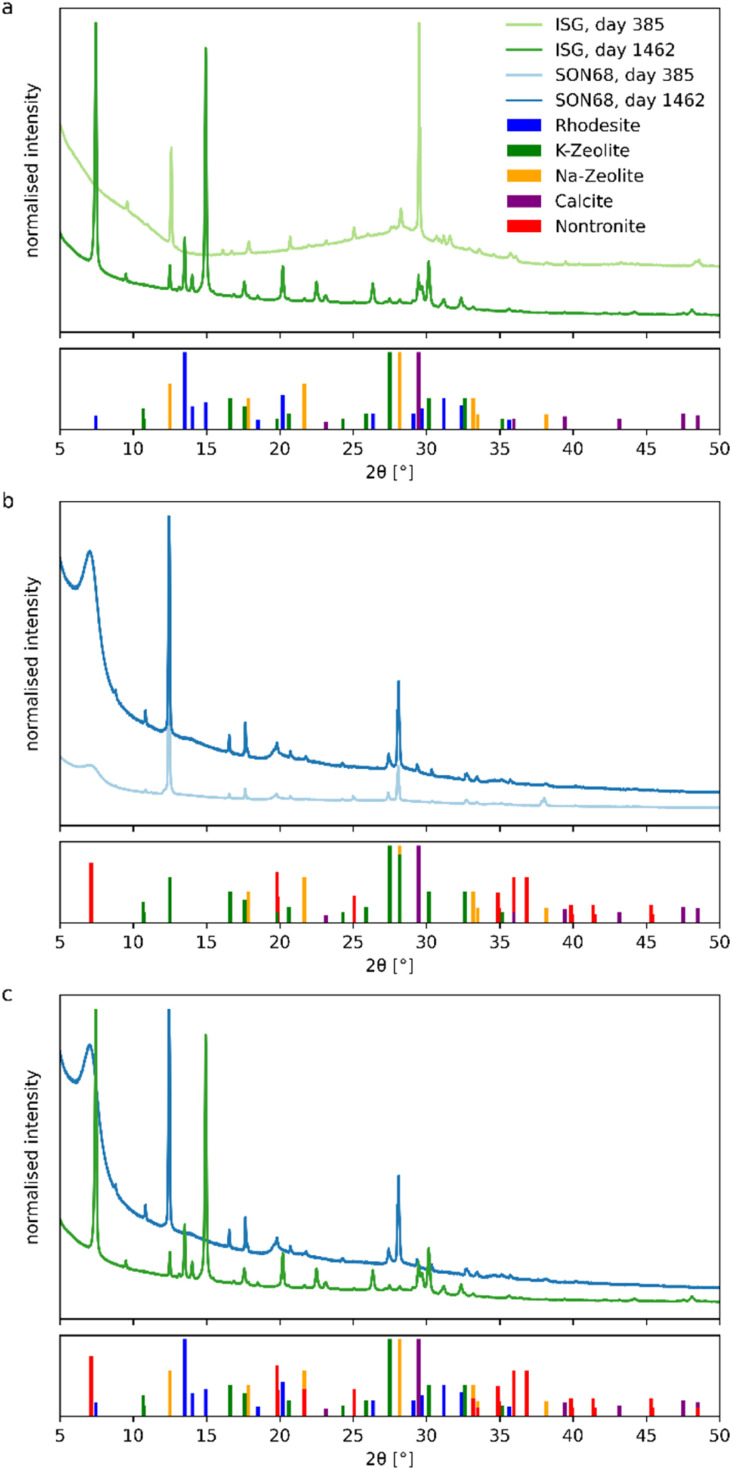
Comparison of XRD pattern of the grain size fraction <5 μm of (a) ISG and (b) SON68 after 385 and 1462 days. (c) Comparison of SON68 and ISG after 1462 days. PDF2-entry numbers: rhodesite 00-081-2027, Na-zeolite 00-012-0214, K-zeolite 00-16-0692, nontronite 00-02-0033, calcite 00-022-1253.

The grain size fraction <5 μm was used for the identification of the secondary phases. For ISG, data provided from Ferrand *et al.*^42^ for day 385 are compared with new data for a sample taken on day 1462 (Fig. 3a). In the sample taken at day 385, mainly Na- and K-zeolite (phillipsite-type) were identified, along with some calcite. Calcite is a drying artefact, because calcite formation during the leaching was unlikely, as the dissolution experiments were performed under inert atmosphere. Although the pH and the element concentrations in solution appeared very stable after day 385, significant changes in the mineralogical composition of the secondary phases were observed in the sample taken after day 1462. There is significantly less broad background of the amorphous glass in the later ISG sample, indicating the dissolution of small glass particles. In addition to the previously identified phases of day 385, rhodesite, a Ca-phyllosilicate, appears as newly formed phase. A decrease of the intensity of the K-zeolite peaks was also noted.

For the SON68 fraction <5 μm, Na- and K-zeolites (phillipsite-type) and calcite were identified. Unlike ISG, SON68 patterns show a diffuse low-angle peak at 2*Θ* = 7°. It belongs to nontronite, a 12.6 Å smectite clay mineral (Fig. 3b), which was identified as a secondary phase. Although such phyllosilicates are difficult to characterize by XRD, Muller *et al.*^47^ showed the presence of nontronite when glasses formulated to immobilize West Valley nuclear waste were leached in product consistency tests in deionized water at 90 °C and 2000 m^−1^. Smectites were also identified by Chave^48^*via* XRD after separation of the alteration products formed on the SON68 glass surface. In a more complex system, *i.e.* glass/iron/claystone the temperature was reported to be a crucial parameter,^49^ as serpentines are observed at 90 °C whereas at 50 °C they are no longer stable^50–52^ and the precipitation of smectites such as nontronite is favored. Nontronite is a typical phase used in geochemical modelling allowing to reproduce both the decrease of the initial alteration rate and the so-called residual regime. In de Combarieu *et al.*,^53^ the SON68 glass dissolution at 90 °C and 80 cm^−1^ in pure water or with iron from the canister and overpack or with argillite was modeled with an affinity law with respect to a nontronite-like phase, which saturation state depends on Si, Al, Fe, Na and Ca activities.

Clay minerals such as saponite or nontronite are also commonly observed as basaltic glass alteration products.^54^ In Parruzot *et al.*^43^ the measure of the interplanar spacing on some of these clayey filaments formed during the corrosion of a synthetic basaltic glass at 90 °C and 100 000 m^−1^ was similar to that in natural palagonites, suggesting that these clay minerals could be di- or trioctahedral smectites such as nontronites or saponites.^55^

In both SON68 samples, no significant amorphous background of the glass was detected. Between day 385 and day 1462 the intensity of the nontronite peaks increased as well as the peak intensities of the other secondary phases. In comparison of the two glass samples of SON68 and ISG taken at the final sampling (Fig. 3c), the two zeolites that formed appear to be similar whereas the third secondary phase – nontronite or rhodesite – formed due to the different initial glass compositions.

Zeolites are typical phases formed when nuclear glasses are altered at alkaline pH values. Depending on the solution and glass composition, on the temperature and pH, different zeolites such as merlinoite, analcime, chabazite, and phillipsite have been identified.^17,21,27,56,57^ The formation of phillipsite, as observed at the glass surface in our study performed in YCWCa, was reported in several glass dissolution studies carried out with the same leaching solution (*e.g.* ref. 58). K-rich zeolite phases were found to be precipitated during soda-lime glass alteration at 50 °C (ref. 59) and ISG alteration at 70 °C and 8280 m^−1^.^25^ In this latter study, rhodesite was also identified by XRD. Unlike previous studies (*e.g.* ref. 25), no resumption of alteration due to secondary phase formation was observed in our study, likely caused by a pH drop below the threshold for alteration resumption due to zeolite formation (pH 10.5 at 90 °C). This threshold was confirmed by studies such as Gin and Mestre^21^ and Fournier *et al.*^17^ Previous work by Muller *et al.*^47^ showed no resumption at pH 9.7 at 90 °C, with resumption occurring above pH 11, linked to phillipsite formation. Similar findings were reported by Neeway *et al.*^60^

#### 3.2.2 General features of the altered glass powders: SEM investigations

**Fig. 4:**
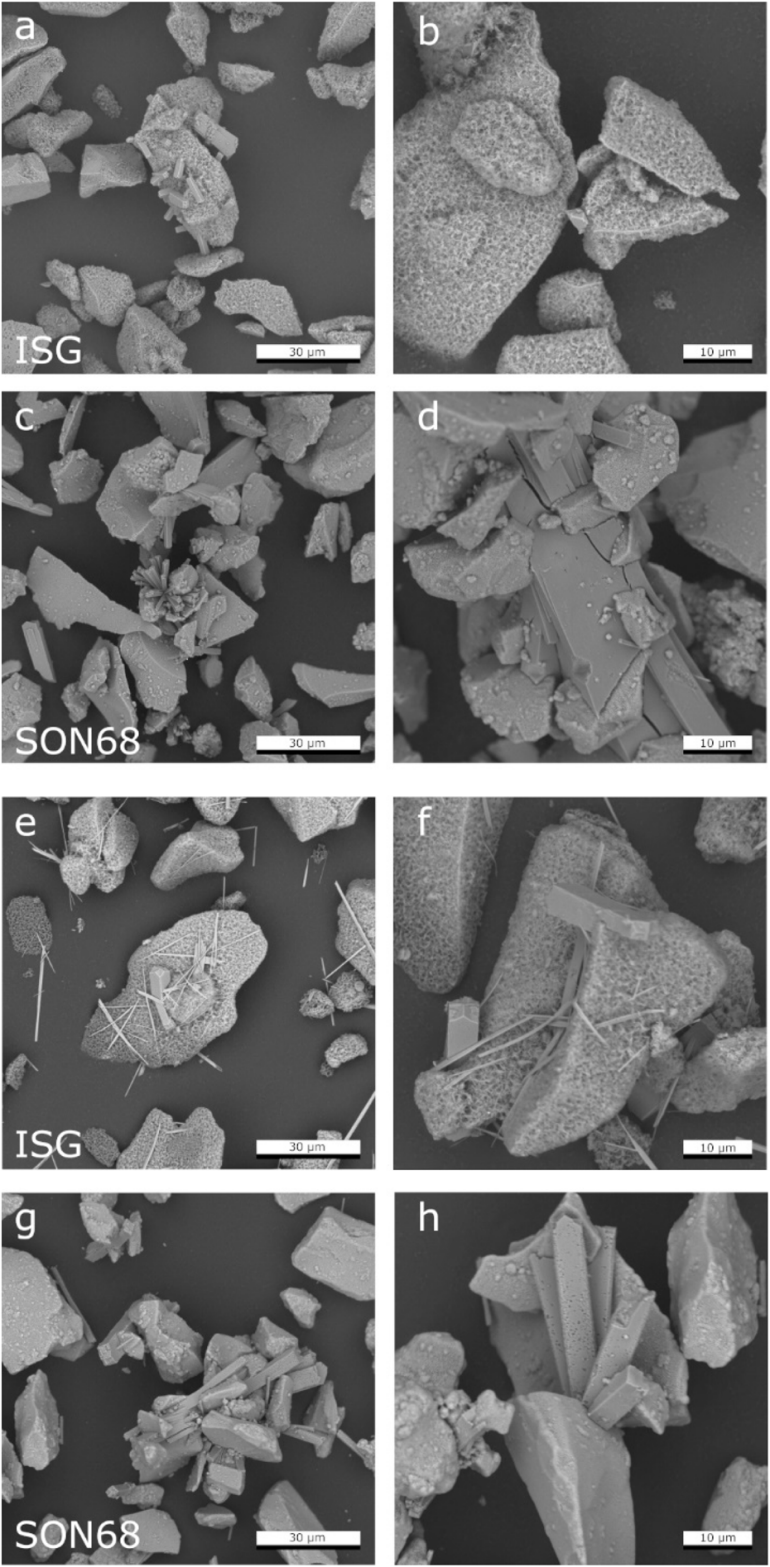
Comparison of SEM images of (a) and (b) ISG after 385 days; (c) and (d) SON68 after 385 days; (e) and (f) ISG after 1462 days (g) and (h) SON68 after 1462 days.

SEM pictures of the powders of ISG and SON68 after 385 and 1462 days of alteration before separation of the <5 μm are presented in Fig. 4. For the ISG sample taken at day 385, in addition to the phases identified *via* XRD, C(A)SH phases precipitating at the surface of the glass particles were identified based on their morphology, as described in Ferrand *et al.*^42^

The precipitation of C(A)SH phases on the glass surface in our study agrees with previous observations on the behaviour of nuclear glasses in alkaline solutions. C(A)SH phases were observed at the surface of ISG after leaching in a KOH solution at pH 11.5 or under different humidities.^30,61^ Under such experimental conditions, these phases were only formed from the Si and Ca released from the glass and not originating from the leaching solution. The presence of C(A)SH phases was also reported with ILW glass corroded in a saturated Ca(OH)_2_ solution,^22,56^ and on ISG after alteration in YCWCa.^25,42^ The presence of C(A)SH phases with a low Ca/Si ratio has also been reported in tests conducted in presence of hardened ordinary Portland cement,^62,63^ which provides Ca needed for the pozzolanic reaction.

**Fig. 5:**
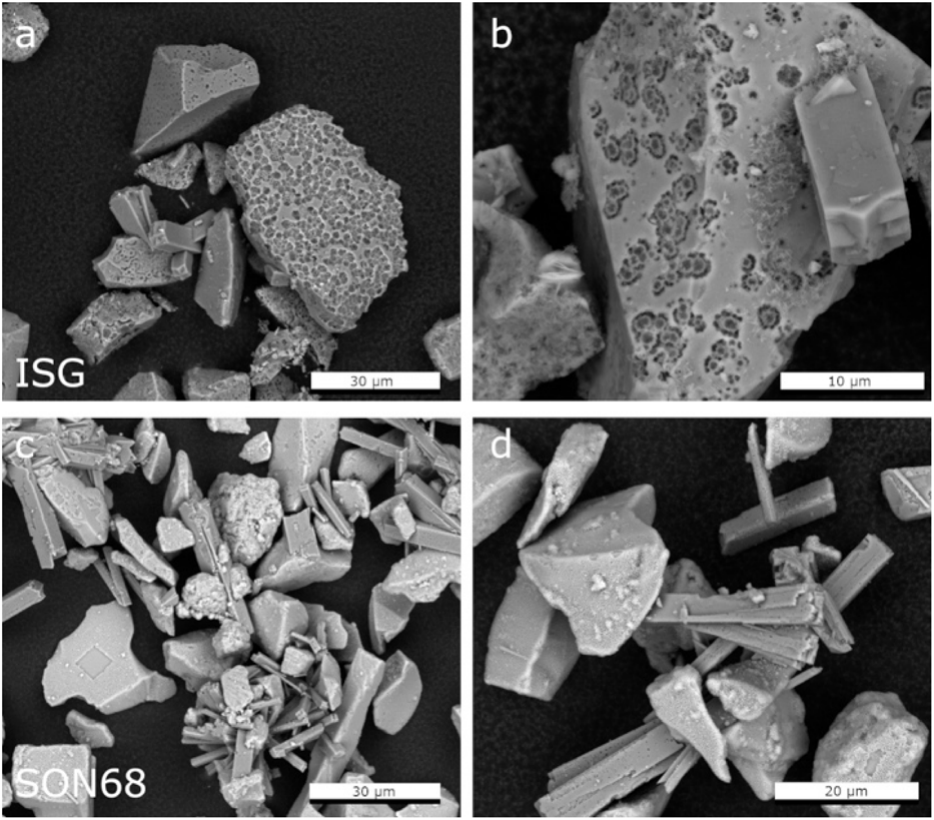
Comparison of SEM images of the separated fraction >5 μm after 1462 days of ISG (a and b) and SON68 (c and d).

The fraction >5 μm allows for investigating the surfaces of the glass particles after removal of the secondary phases (Fig. 5). Confirming the findings of Ferrand *et al.*,^42^ the presence of spherical pores in the pristine ISG was also observed at day 385, indicating areas of preferential dissolution. These were again observed at day 1462, but appear more prominent in the latter sample (Fig. 5a and b). The presence of pits in the glass surfaces after removal of the secondary phases could be attributed to presence of percolation channels in the medium range structural order of the glass and of high alkali concentrated regions, which could lead to a local pH increase in the solution trapped at the interface between the SAL and the pristine glass after alkali release.^64,65^

The zeolites in this ISG sample exhibit the typical rod-shaped, rectangular morphology (Fig. 4a). The zeolites of the SON68 sample appeared as aggregates already at day 385 (Fig. 4c and d). Some very large zeolites had formed between glass particles, *i.e.* appear to have grown in the inter-particle space formed by the SON68 glass (Fig. 4d). The maximum crystal size of the zeolites in the SON68 samples was observed for day 952, with lengths of up to 100 μm. The SON68 sample taken at day 1462 still contained zeolites, but with a porous surface and a smaller grain size than the zeolites observed in earlier samples (Fig. 4g and h). This observation indicates that with time some zeolites in these experiments became unstable and started dissolving due to the decreasing pH. The glass surfaces of the grain size fraction >5 μm of SON68 do not show the spherical pores in the pristine glass as observed for ISG (Fig. 5c and d). No areas of preferential dissolution were observed. In contrast to SON68, in ISG, a new phase occurred at the later stage of the experiments, which was characterized by needle-like crystals with length of 10 to 30 μm on day 1462 (Fig. 4e and f). This is the typical morphology of the mineral rhodesite, which was already identified by XRD. The general morphology of the secondary silicate minerals with well-developed habitus indicates crystal growth at low to moderate supersaturation and were predicted to form for the ISG glass in Ferrand *et al.*,^42^ based on the solution composition.

#### 3.2.3 The interface of the dissolving glass and SAL: TEM analyses

**Fig. 6:**
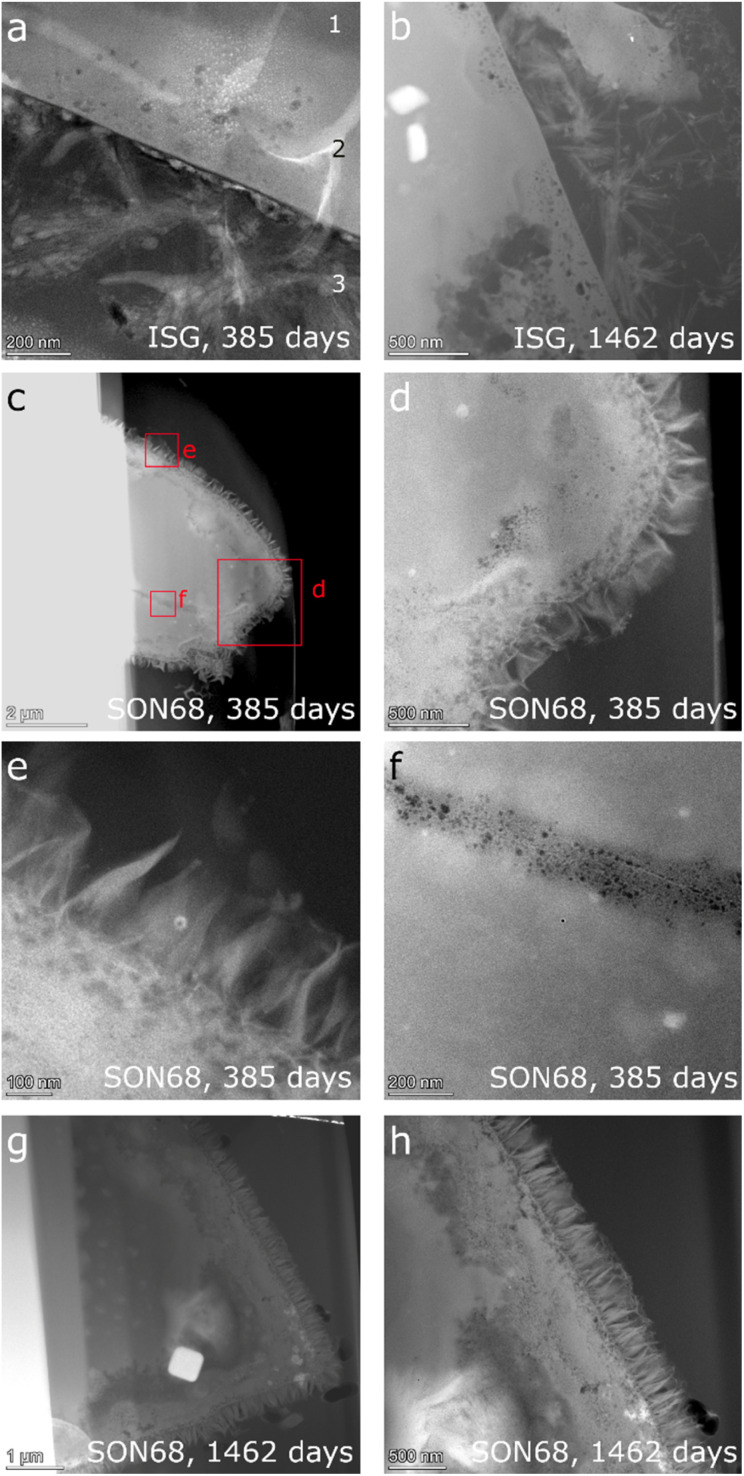
HAADF STEM images of ISG (a) at day 385 (“1” indicates the pristine glass, “2” the porous layer and “3” the secondary phases) (b) at day 1462, and (c)–(f) SON68 at day 385 and (g and h) at day 1462.

For comparability, a new thin TEM lamella sample of ISG glass after 385 days (Fig. 6a) was prepared in addition to the one already examined by Ferrand *et al.*^42^ STEM images of this lamella are shown in comparison to those acquired from the lamella of ISG of day 1462 (Fig. 6b) and with two corresponding SON68 lamellae of day 385 (Fig. 6c–f) and 1462 (Fig. 6g and h). In addition, lamellae of pristine ISG and SON68 were prepared as reference samples and respective STEM images are available in the data publication.^44^

The observations of Ferrand *et al.*^42^ for ISG of day 385 (Fig. 6a) were confirmed. Three zones were identified, (1) pristine glass, (2) porous altered layer (SAL), and (3) secondary phases at the glass surface (Fig. 6a). The pores within the SAL (2) were visible as dark round features and between 10 and 50 nm in size, contained no solids and were not connected. The porous altered layer itself had a thickness between 80 and 250 nm. The very bright elongated features in the image are an artefact which is due to Na being mobilized and redeposited during the TEM measurement. This effect of irradiation damage by the electron beam was already observed during the TEM investigations of the reference glass samples. C(A)SH phases appear to grow from the porous layer of ISG.^42^ The FIB-lamella of ISG taken on day 1462 (Fig. 6b) showed a SAL and secondary phases similar to those observed on day 385 (Fig. 6a). Only details changed *e.g.*, the crystal habitus of the C(A)SH phases which appeared to grow longer and thicker with time. The typical thickness of the porous layer increased slightly up to 300 nm. The general structure of the SON68 glass alteration layer after 385 days has similar features to those of ISG (Fig. 6c–f), including a porous altered layer (SAL) with secondary phases growing from it. Instead of the C(A)SH phases of the ISG sample, on SON68 clay minerals identified as nontronite by XRD have grown from the glass alteration layer. Sometimes they originated from porous layer, with a typical fibrous structure and lengths of about 200 nm to 300 nm (Fig. 6e). After 385 days, the thickness of the SON68 porous layer was heterogeneous, ranging from 250 nm to 750 nm. A crack in the glass was also recognized, which was filled with porous altered glass (Fig. 6f). Between day 385 and day 1462, the general structure of the porous layer and secondary phases remained the same. However, the nontronite layer became denser and the typical size of the nontronite increased slightly up to 300–350 nm, in good agreement with the increase of the XRD peak intensities (see 3.2.1). The thickness of the porous layer of the SON68 sample at day 1462 was in the range of 700 to 1200 nm and contained more pores than the one of day 385.

**Fig. 7:**
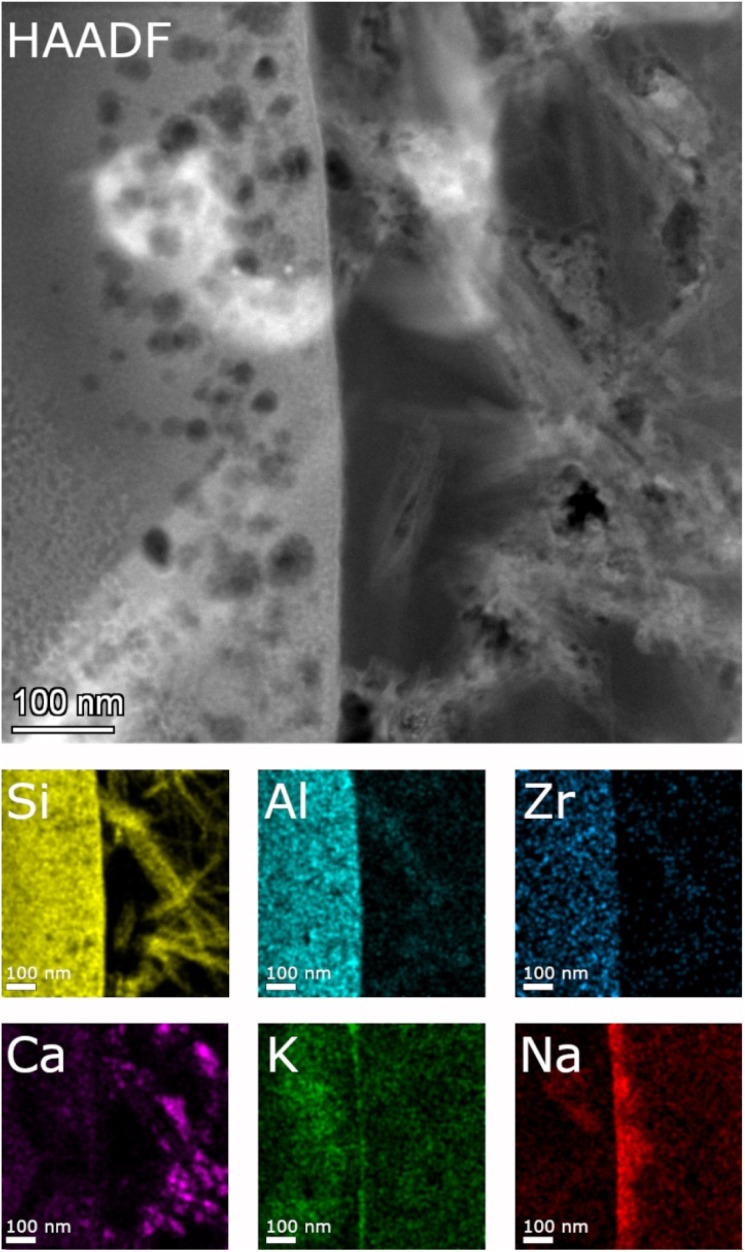
STEM EDS mappings of the alteration zone of ISG after 385 days as observed in the HAADF STEM image. Elemental mappings of Si–K, Al–K, Zr–K, Ca–K, K–K, and Na–K lines.

**Fig. 8:**
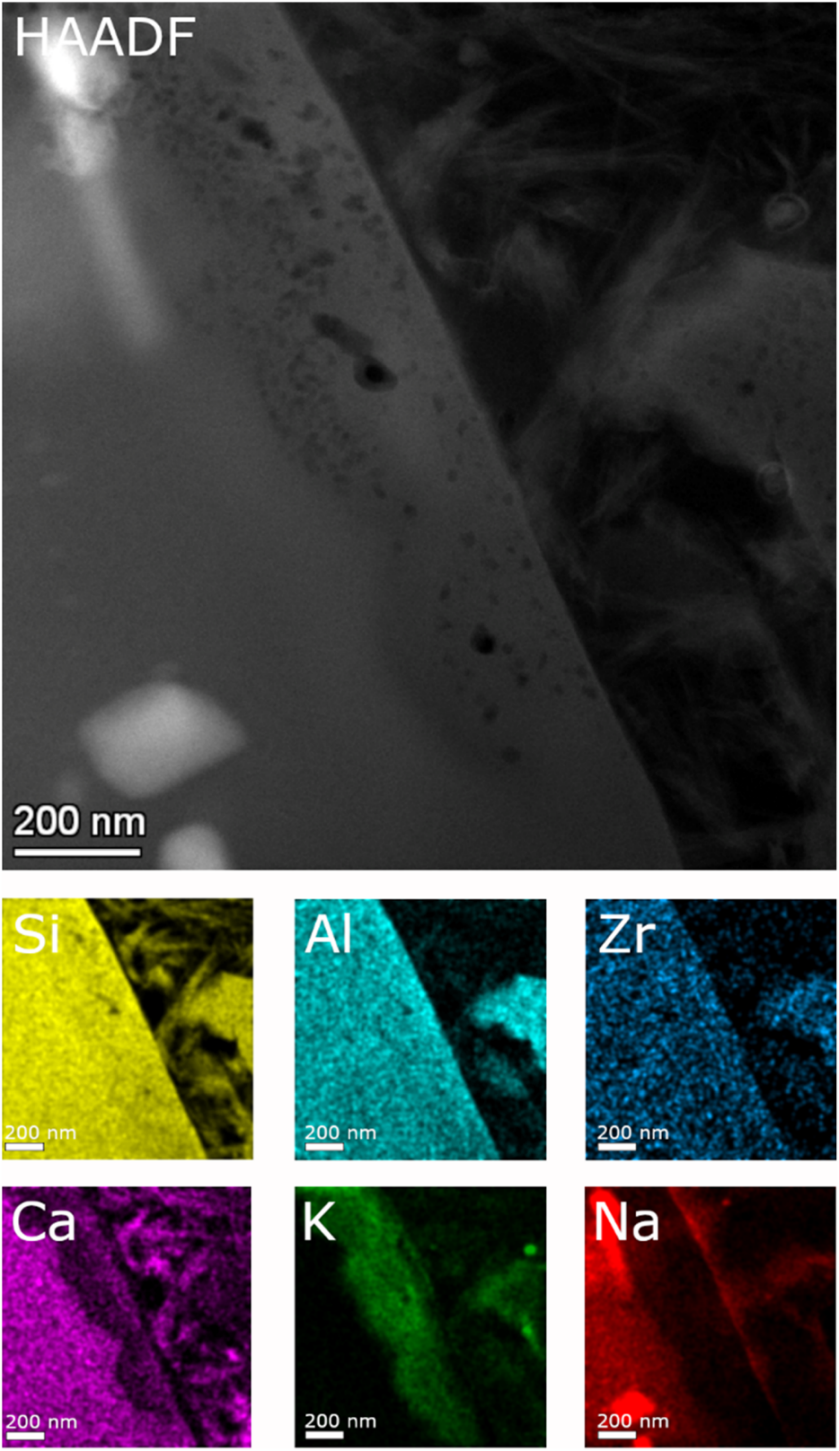
STEM EDS mappings of the alteration zone of ISG after 1462 days as observed in the HAADF STEM image. Elemental mappings of Si–K, Al–K, Zr–K, Ca–K, K–K, and Na–K lines.

**Fig. 9:**
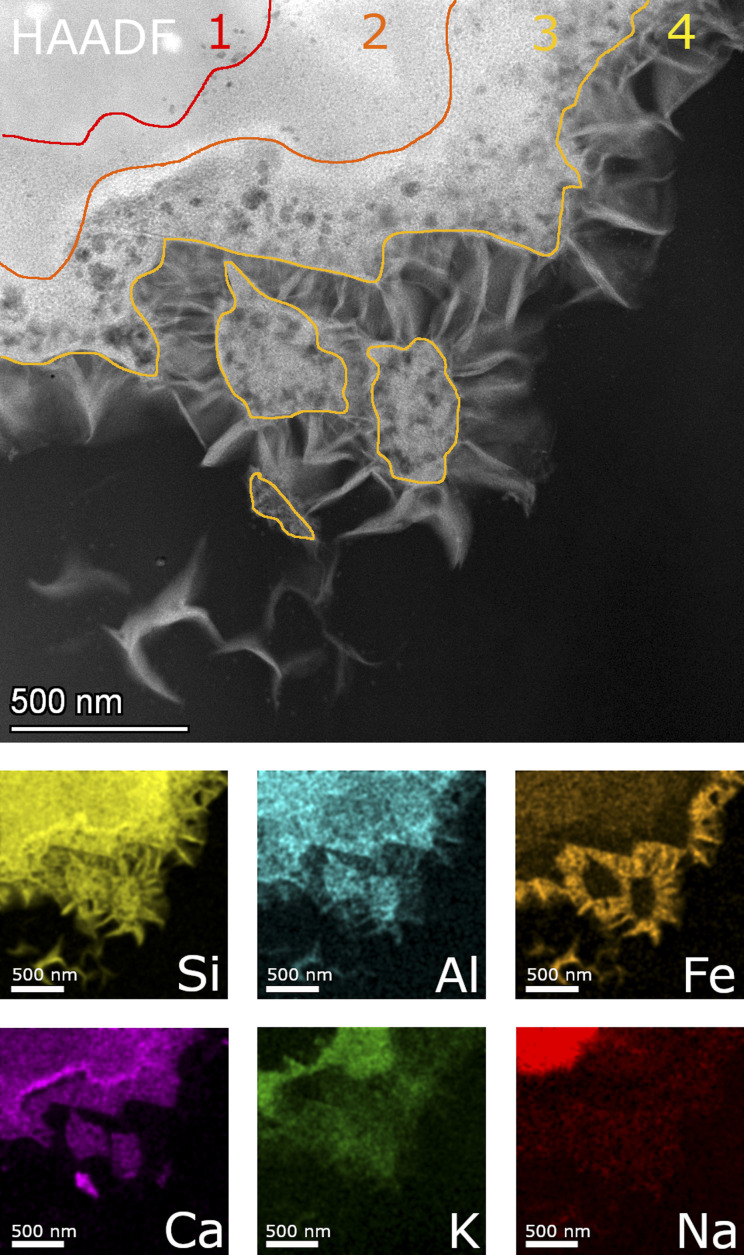
STEM EDS mappings of the alteration zone of SON68 after 385 days as observed in the HAADF STEM image. Elemental mappings of Si–K, Al–K, Fe–K, K–K, Ca–K and Na–K lines. Lines depicting the different zones are only guiding lines, mainly based on the mappings of Fe, K and Na.

Detailed TEM-EDS element mappings were taken across the interface between the porous layer and secondary phases on the ISG (Fig. 7 and 8) as well as on the SON68 FIB sections after day 385 and day 1462 (Fig. 9). The Si, Al, Ca mappings confirmed the presence of secondary calcium (aluminum) silicate hydrate phases (C(A)SH) at the glass surface. The mappings of Na and K indicate the uptake of K into the porous layer as well as an enrichment of K at the interface between the porous layer and the C(A)SH phases. Na is enriched at the same interface as well, but the enrichment is located on the solution side of the K enrichment, whereas very little Na remained in the porous layer. The general observations for ISG of day 1462 (Fig. 8) are similar. The exchange of Na by K is now clearly visible, leading to a depletion of Na and an enrichment of K in the porous layer. The porous layer was about 200–400 nm thick, based on the K mapping and the observation of pores in the HAADF image (Fig. 8).

Fig. 9 shows a detailed HAADF image of the SON68 glass alteration layer at day 385. The apparent structure of SAL of SON68 is more complex than that of ISG, consisting of larger pores immediately at the interface with the secondary phases, and finer pores towards the apparently pristine glass. The HAADF images and the EDS mapping of this region reveal four zones which differ in structure and composition, (1) apparently pristine glass, (2) amorphous layer with fine pores, (3) a layer with coarse pores, and (4) secondary phases. The pristine glass can be recognized due to the presence of Na which is depleted in the other zones, whereas no K has been taken up from the solution. A homogeneous distribution of Al, Si, Ca is also typical for the pristine glass. Zone 2 of SON68 is characterized by fine pores and contains the highest concentration of K, compared to the other zones. At the interface between zone 2 and zone 3, an enrichment of Ca can be noted. In addition, zone 3 also contains K, but at a lower K/Si ratio than zone 2. In comparison to zone 1 (pristine glass) and zone 2, zone 3 is depleted in Fe. The secondary phases (zone 4) contain Fe, Si, Al as well as Mn and Zn (mappings of Mn and Zn: data publication ‘SON68 day 385 TEM-EDS Maps’^44^). This is in good agreement with the mineral nontronite as secondary phase as identified in the XRD patterns of the SON68 sample after 385 days (Section 3.2.1).

**Fig. 10:**
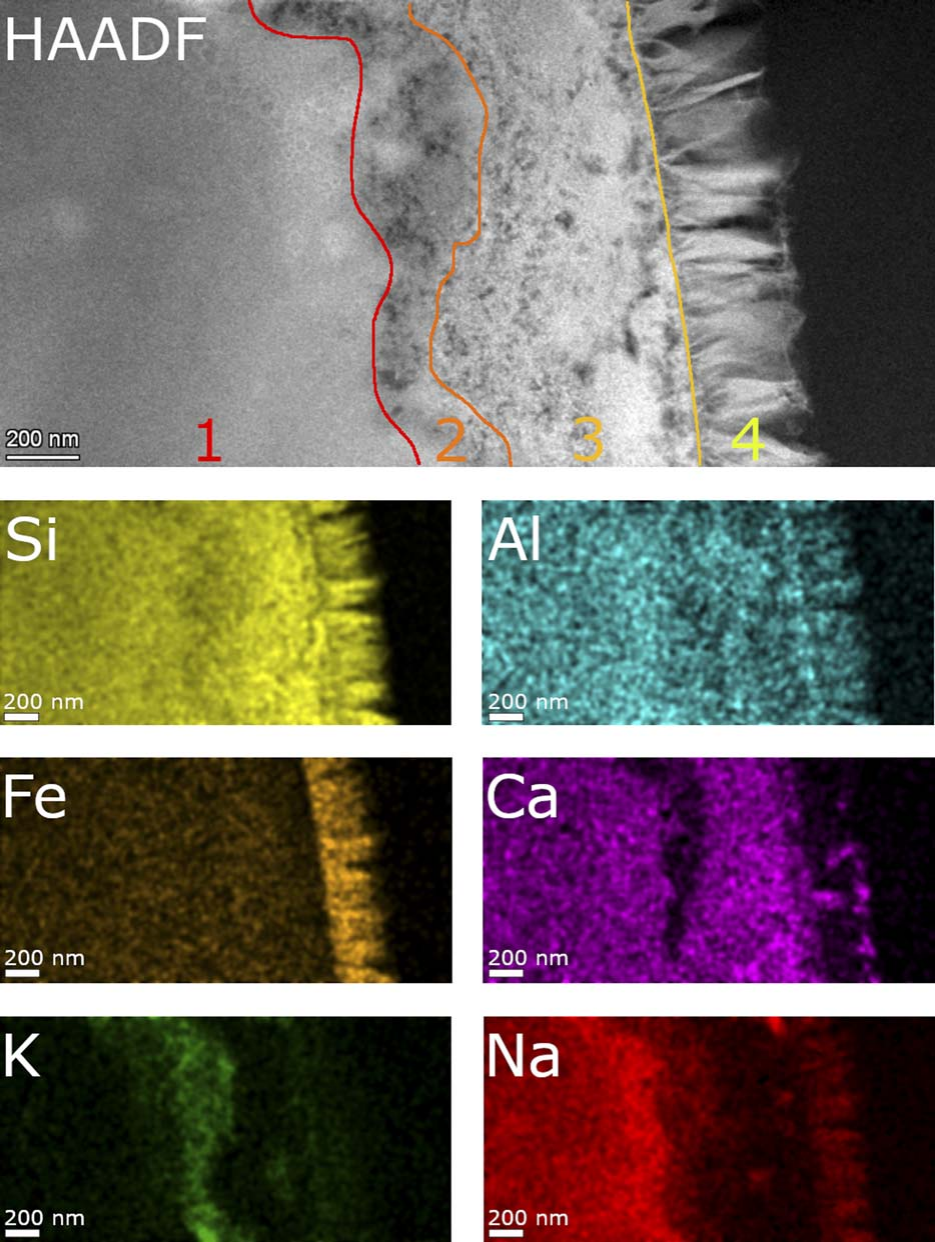
STEM EDS mappings of the alteration zone of SON68 after 1462 days as observed in the HAADF STEM image. Elemental mappings of Si–K, Al–K, Fe–K, K–K, Ca–K and Na–K lines.

SON68 glass taken at day 1462 (Fig. 10) is still characterized by the four zones described above. In contrast with the earlier sample, Ca was also detected in the nontronite layer, apparently in exchange for K.

In summary, the combined observations of the evolution of pH and element concentrations in solution, XRD and electron microscopy point to a leaching process in which the leached layer(s) form a reactive barrier. A colloidal gel layer, as observed at lower SA/V, was not formed due to the formation of secondary phases. However, a rate drop towards a residual dissolution rate regime can be observed for both glass types, which establishes itself earlier for ISG than for SON68 glass. Since no colloidal SAL can be responsible for the rate drop, the water diffusion and ion exchange kinetics may be responsible for the slowdown in the glass dissolution kinetics, in combination with the drop in pH. The exchange of K with Na as well as the Ca and Fe release out of the respective glass types are indicators for such an interdiffusive leaching reaction. Such a very thin SAL has been described earlier by Grambow and Müller^19^ and by Jégou *et al.*^66^ Grambow and Müller^19^ describe a diffusion barrier which “can be considered as part of the glass phase” as a condition for a model to simulate nuclear waste glass dissolution. This condition appears to be fulfilled between day 385 and day 1462 for both glass types. The leaching process of SON68 is more far reaching into the solid and creates a more complex microstructure. The leaching of Fe from SON68 and Ca from ISG indicates that the nature and type of the secondary phase which forms directly at the interface has a significant impact upon the leaching process. At the same time, the more mobile alkalis behave similar in both glasses, leading to an uptake of K into the alteration layer and a release of Na out of it (Fig. 10).

## 4 Conclusions

Long-term dissolution experiments, spanning almost 1500 days, were conducted with two types of glass at high pH, very high SA/V ratio, and 70 °C. Analysis of solution composition and changes in glass and alteration products provided insights into the behaviour of nuclear waste glasses under disposal relevant conditions. Boron release (NL(B)) indicated a rapid initial dissolution, followed by a slowing down of the dissolution, related to a significant pH drop. ISG glass reached a residual rate regime, while SON68 glass only approached it near the experiment's end. SON68 showed a higher total amount of dissolved glass at the end of the experiments (0.78 g m^−2^) compared to ISG (0.44 g m^−2^). However, during the final phases of the experiments, the dissolution rates of the two glass types are in a very similar order of magnitude.

Secondary phases were identified *via* XRD, revealing that clay minerals formed on SON68, while C(A)SH phases and at later stages rhodesite appeared on ISG. Nontronite formed on SON68 due to its iron content, which ISG lacks. EDS and SEM analyses of the secondary phillipsite-type zeolites showed similar mineralogical compositions and structures in both glass types.

**Fig. 11:**
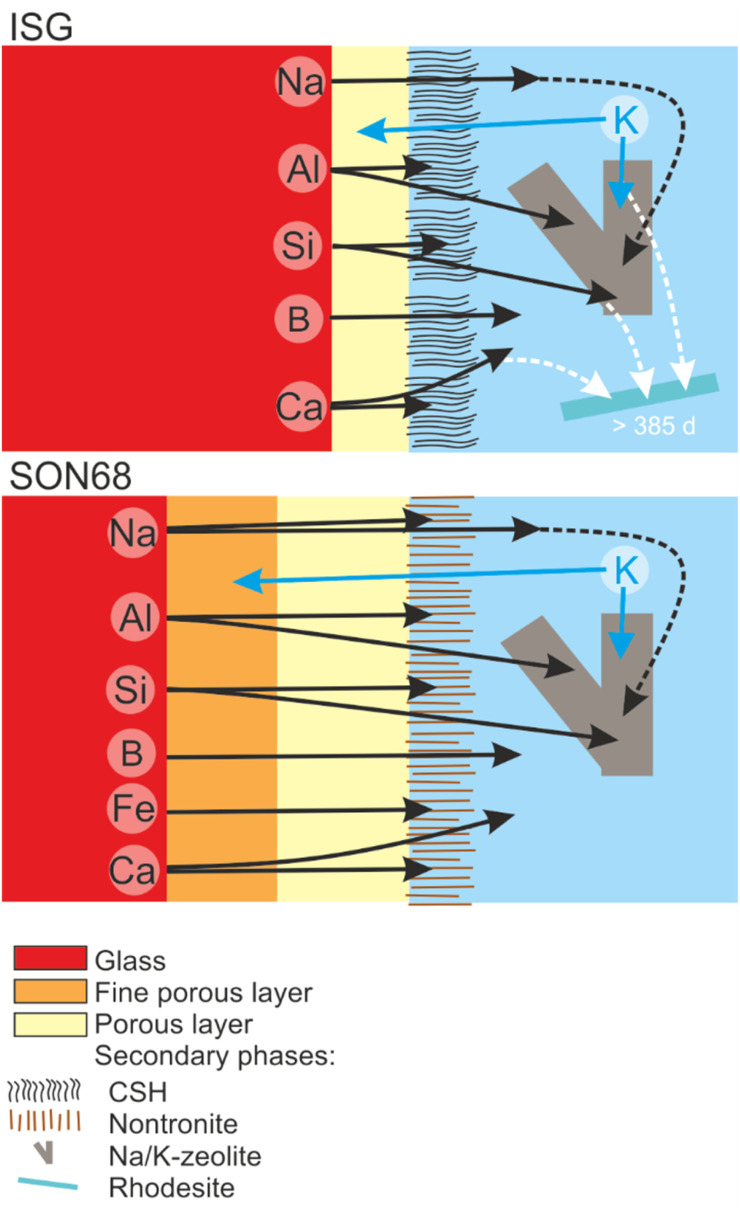
Sketch summarizing the evolution of glass alteration from day 385 to day 1462 for ISG and SON68.

TEM observations of the SAL near the pristine glass revealed a porous, foam-like structure, contrasting with the usual colloidal SAL seen at alkaline pH and low SA/V. The detailed TEM-EDS observations showed the exchange of K for Na and Ca, as well as the depletion of iron from the SAL of SON68 which then forms nontronite. The SAL nanostructure of SON68 is more complex compared to ISG, consisting of two zones with varying porosity in contrast to only one zone observed in ISG. These layers may have hindered water transfer and glass constituent release, in addition to the positive effect of the pH drop, which decreases the hydrolysis of the silica network. The presence of secondary phases at the SAL-solution interface did not destabilize the SAL. No resumption of alteration due to secondary phase formation was observed, likely also caused by the pH drop below the threshold for alteration resumption due to zeolite formation. Fig. 11 and the graphical abstract summarize the different processes during the glass dissolution as constructed from the individual observations, including ion exchange at the dissolving glass surface, SAL formation and the precipitation of secondary phases at the SAL – aqueous solution interface as well as in the aqueous solution.

In conclusion, the combination of alkaline conditions and very high reaction progress does not lead to the dissolution of the glass by a dissolution-reprecipitation mechanism, as typically observed at much lower SA/V ratios, probably because the pH decreases quickly to the moderately alkaline domain where diffusion is the driving mechanism. The formation of secondary phases, depending on the glass composition, is favoured for both glasses SON68 and ISG. Already within 1 year the glass particles are covered by a precipitated layer of secondary phases, on top of a very thin, porous surface alteration layer. Afterwards the secondary phases continue to grow. At the relatively mildly alkaline pH reached within the first year of the experiments the growth of secondary phases is not the rate controlling process, but the diffusion of cations through the SAL. The long duration of the experiments has allowed to reach or approach a residual rate regime and has given a precise description of the glass alteration layer for periods exceeding most experimental time frames found in literature. The experiments at very high SA/V show that for the borosilicate glasses SON68 and ISG, confined disposal conditions can lead to a pH decrease that annihilates the deleterious effect of a high initial pH, even for young cement water. Whether or how fast such conditions can be reached will depend on the precise glass composition, local SA/V conditions and the transport properties of the concrete.

## Data availability

Data for this article, including results of long-term static dissolution experiments with data of pH evolution as a function of time, element release normalized mass losses of several elements and microscopic data is available at Jülich DATA at https://doi.org/10.26165/JUELICH-DATA/HC3IOI.

## Author contributions

Conceptualization: F. B. (lead), K. F.; methodology: K. F., M. K., J. B. K. L., investigation: K. F., S. C., M. K., J. B.; writing – original draft preparation: F. B., M. K., K. F.; writing – review and editing: all authors; funding acquisition: D. B., K. L.

## Conflicts of interest

There are no conflicts to declare.
